# Exercise and Escitalopram in the Treatment of Anxiety in Patients with Coronary Heart Disease: One Year Follow-Up of the UNWIND Randomized Clinical Trial

**DOI:** 10.3390/jcdd9100320

**Published:** 2022-09-22

**Authors:** James A. Blumenthal, Patrick J. Smith, Wei Jiang, Alan Hinderliter, Lana L. Watkins, Benson M. Hoffman, William E. Kraus, Stephanie Mabe, Lawrence Liao, Jonathan Davidson, Andrew Sherwood

**Affiliations:** 1 Department of Psychiatry and Behavioral Sciences, Duke University Medical Center, Durham, NC 27710, USA; 2 Department of Medicine, University of North Carolina at Chapel Hill, Chapel Hill, NC 27710, USA; 3 Department of Medicine, Duke University Medical Center, Durham, NC 27710, USA

**Keywords:** anxiety, depression, exercise, escitalopram, coronary heart disease

## Abstract

Anxiety is common among patients with coronary heart disease (CHD) and is associated with a worse prognosis. UNWIND was a 12-week randomized clinical trial comparing exercise and escitalopram to placebo on measures of anxiety, depression, and CHD biomarkers. Primary results of the trial reported that treatment with escitalopram, but not exercise, was associated with significant reductions in anxiety and depression. At 1-year follow-up, participants completed the Hospital Anxiety-Depression Scale-Anxiety (HADS-A) along with the HADS-Depression (HADS-D), the Beck Depression Inventory-II (BDI-II), and the Godin Leisure Time Exercise survey to assess physical activity. Results showed that those patients randomized to escitalopram had lower scores on the HADS-A compared to those randomized to exercise (*P* = 0.006) and had less depression compared to exercise on the HADS-D (*P* = 0.004) and BDI-II (*P* = 0.004). Participants randomized to exercise reported higher levels of physical activity at 1-year compared to those randomized to Placebo (*P* = 0.039). However, despite reporting being more physically active, those randomized to exercise did not have less anxiety or depression compared to placebo controls. Escitalopram appears to be a safe and effective treatment for anxiety; exercise has many health benefits, but does not appear to be effective in treating anxiety.

## 1. Introduction

Anxiety is the most common psychiatric disorder in the United States, affecting more than 40 million adults. Almost 1 in 5 Americans suffered from a diagnosed anxiety disorder in the past year [1] and as many as a third of U.S. adults experience any anxiety disorder at some time in their lifetimes [2]. Anxiety also is highly prevalent in cardiac populations, with estimates ranging from 25% to 44% [3]. In addition, there is growing evidence that anxiety is an independent predictor of worse outcomes in both healthy adults and in cardiac populations [4,5,6,7,8,9,10,11,12]. For example, Frasure-Smith and colleagues [7] reported that more than 40% of post-myocardial infarction patients had elevated symptoms of anxiety and that anxiety was associated with more than a 50% increased risk for major adverse cardiac events over a 2-year follow-up. Strik and colleagues [13] found that while symptoms of depression and anxiety both were associated with increased cardiac events over an average follow-up period of 3.4 years, anxiety was an independent predictor of adverse events, and further noted that anxiety actually explained away the relationship between depressive symptoms and worse prognosis.

Although there have been a number of randomized clinical trials evaluating the treatment of depression in patients with CHD [14,15,16], we are not aware of any studies that have targeted anxiety in CHD patients. The UNWIND trial compared the effects of escitalopram and exercise to a pill placebo in patients with stable CHD. The primary endpoint was the Anxiety subscale on the Hospital Anxiety and Depression Scale (HADS-A). Results showed that escitalopram, but not exercise, reduced anxiety more than placebo [17], and that these benefits were maintained over 6-months [18]. To examine the longer-term benefit, the present report extends the follow-up over a 1-year interval.

## 2. Methods

### 2.1. Trial Overview

UNWIND was a single-site, parallel group randomized clinical trial designed to evaluate the effects of aerobic exercise and escitalopram compared to a placebo on anxiety symptoms and CHD biomarkers among anxious individuals with stable CHD. Enrollment began in January 2016 and the interventions were completed in May 2020. The trial was approved by the institutional review board at Duke University Medical Center, and written informed consent was obtained from all participants. Follow-up assessments were performed 1 year after completion of the 12-week interventions; the last assessment was performed in December 2021. The study is registered at www.clinicaltrials.gov (ID: NCT02516332).

### 2.2. Participants

In total, 128 sedentary men and women with CHD and an anxiety symptom severity score of ≥ 8 on the anxiety subscale of the Hospital Anxiety and Depression Scale (HADS-A) [19] and/or a DSM-5 primary diagnosis of an Anxiety Disorder were enrolled in the trial. Patients with a primary psychiatric diagnosis other than an anxiety disorder or who were receiving treatment for a psychiatric disorder were excluded. Demographic and clinical characteristics are summarized in Table 1.

### 2.3. Treatment Conditions

**Aerobic Exercise.** Patients exercised three times per week under medical supervision at a designated cardiac rehabilitation facility in central North Carolina. Exercise intensity was aimed at a level of 70–85% of participants’ VO_2peak_ as determined at the time of their baseline exercise treadmill test. The exercise protocol consisted of 10 min of gradual warm-up exercises followed by 35 min of continuous walking, biking, or jogging, and 5 min of cool down exercises. Patients were instructed to monitor their radial pulses and heart rates were checked three times per session to ensure that participants were within their prescribed exercise training ranges.

**Escitalopram/Placebo Pill.** Participants met with the treating psychiatrist, blinded to treatment condition, at weeks 1, 2, 4, 8, and 12 for medication adjustment. All participants started on 5 mg once per day and were titrated up to a maximum daily dose of 20 mg or placebo equivalent at week 4. 

No treatment-related adverse effects were noted during the trial. At the end of the intervention period, participants were given the option of receiving a prescription for escitalopram and/or a prescription for a home-based exercise program based upon a post-treatment exercise treadmill test. Test results were provided to patients’ primary care physicians for follow-up care as needed.

### 2.4. One-Year Follow-Up Assessments

During the follow-up period, participants were surveyed for any post-intervention psychiatric treatments and current levels of physical activity; participants also completed a battery of questionnaires to assess anxiety, depression, and stress.

**Physical activity.** Physical activity was quantified by the Godin Leisure Time Exercise Questionnaire [20]. 

**Anxiety.** The primary endpoint was the score on the 14-item Hospital Anxiety and Depression Scale (HADS) [19]. Additional anxiety assessments included the 20-item Spielberger State-Trait Anxiety Inventory-Trait (STAI) [21] and the Generalized Anxiety Disorder 7-item questionnaire (GAD-7) [22].

**Depression.** Depressive symptoms were assessed by the Beck Depression Inventory-II (BDI-II) [23] and the HADS-D [19].

**Perceived Stress**. Perceived stress was measured by the Perceived Stress Scale (PSS) [24], a 10-item survey that taps the degree to which individuals feel that events in their lives are unpredictable and uncontrollable.

### 2.5. Data Analysis

Data were analyzed using SAS version 9.4 (Cary, NC, USA). Differences between those lost to follow-up and those participants who were available for 1-year follow-up assessments were compared using chi-square analyses for categorical data and *t*-tests for continuous variables. For analyses of treatment group differences in anxiety, depression, and stress measures at follow-up, multiple regression analyses were used with 1-year outcomes serving as the outcome variables controlling for the pre-treatment level of the respective outcome, age, sex, race, baseline diagnosis of anxiety disorder, history of myocardial infarction, and treatment group as the predictor of interest. Parallel analyses were conducted for HADS-A, the primary endpoint, and for the STAI, and GAD-7 along with depression and stress measures. In order to delineate planned group contrasts and to account for potential differential treatment patterns following post-treatment assessments, we first examined preplanned contrasts and conducted unadjusted follow-up comparisons of individual group differences. Two pre-planned contrasts were conducted, including a comparison of (1) the two active treatment groups (Escitalopram and Exercise) to Placebo and (2) Escitalopram versus Exercise. Multiple imputation was used to account for missing data, including enrolled participants who did not complete follow-up assessments. Assumptions regarding linearity, additivity, and independence were assessed and found to be acceptable prior to analysis.

## 3. Results

Of the 128 participants initially randomized, 86 (67%) were able to provide 1-year follow-up data. Participants who completed follow-up were evenly distributed across groups, with 35 participants in Exercise (67%), 35 in Escitalopram (66%), and 16 in Placebo (70%) completing 1-year assessments (*P* = 0.956). Participants lost to follow-up were more likely to be female (46% vs. 27%, *P* = 0.044) and tended to have a clinical anxiety diagnosis at baseline (38% vs. 20%, *P* = 0.058).

### 3.1. Treatments Received during the One-Year Follow-up Period

Participants were asked to report any psychiatric treatments that they received during the 1-year post-intervention follow-up period, including participation in counseling/psychotherapy, anxiolytic medication use, and use of herbal medications with potential psychotropic properties. Twenty participants (24%) reported taking anxiolytic medications (14 escitalopram, 2 bupropion, and 1 each for fluoxetine, buspirone, alprazolam and clonazepam), including 13 (37%) participants in Escitalopram, 6 (17%) in Exercise, and 1 (6%) in Placebo (chi-square = 6.90, *P* = 0.032). In addition, 3 participants reported receiving counseling or psychotherapy (2 participants in Exercise and 1 in Escitalopram). 

### 3.2. Physical Activity

Self-reported physical activity obtained from the Godin Questionnaire revealed that the overall sample reported engaging in 2.9 (SD = 2.6) bouts of moderate-to-vigorous physical activity per week, corresponding to a median of 63 mins/week (IQR: 0, 150), with levels of 100 mins/week (IQR: 30, 180) in Exercise, 60 mins/week (IQR: 0, 110) in Escitalopram, and 30 mins/week (IQR: 0, 100) in Placebo. Adjusted group comparisons revealed that the active treatment conditions tended to exhibit greater physical activity levels compared to Placebo (*P* = 0.087), with participants randomized to Exercise reporting higher levels of physical activity at 1 year compared to those randomized to Placebo (*P* = 0.039) and a non-significant trend for greater activity compared with Escitalopram participants (*P* = 0.143)

### 3.3. Anxiety

**Figure 1:**
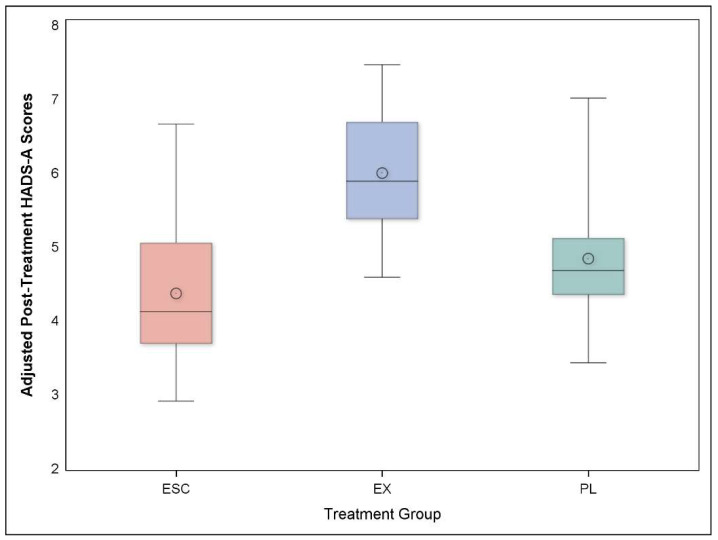
Comparison of scores on the HADS-A by treatment group at one year. Adjusted HADS-A anxiety scores at 1-year follow-up revealed that participants in the Escitalopram condition (ESC) had lower scores compared to Exercise (EX) (*P* = 0.006). Multiple regression analyses were used with 1-year HADS-A scores serving as the outcome variable controlling for the pre-treatment HADS-A score, age, sex, race, baseline diagnosis of anxiety disorder, history of myocardial infarction, and treatment group as the predictor of interest.

Examination of changes in HADS-A scores revealed that the Escitalopram group had less anxiety compared to either Exercise or Placebo groups; the average HADS-A score for Escitalopram was 4.5 (3.7, 5.3) compared to 5.6 (4.8, 6.5) for Exercise, and 5.2 (3.9, 6.4) for Placebo. Examination of pre-planned contrasts revealed no significant differences between both active treatment groups versus Placebo (*P* = 0.628) but lower scores for Escitalopram compared to Exercise (*P* = 0.006) (Figure 1). Because patients randomized to Escitalopram were more likely to receive escitalopram or other anxiolytic medications after completing the 12-week intervention, the longer-term effects of the intervention are confounded by ongoing psychotropic medication use. Therefore, in a new analysis, we eliminated any patients receiving intervening treatments during the 1-year follow-up period. When those participants who were taking anxiolytic medications during the follow-up period were removed from the analysis, those initially randomized to Escitalopram, but who discontinued the medication at the conclusion of the 12-week intervention, continued to report less anxiety compared to those randomized to Exercise (*P* = 0.052).

Results of the ancillary measures of anxiety largely paralleled results from the primary outcome. Although the active treatment groups were not different from placebo controls on the STAI (*P* = 0.455), STAI scores for Escitalopram group were lower compared to Exercise (*P* = 0.006) and tended to be lower compared to Placebo (*P* = 0.083). Treatment group scores for the STAI were 32.8 (30.7, 34.9) for Escitalopram, 36.3 (34.0, 38.6) for Exercise, and 37.4 (34.2, 40.5) for Placebo. A similar pattern was observed for the GAD-7, with no significant differences between active treatment groups and Placebo (*P* = 0.446), but lower anxiety scores for Escitalopram compared to Exercise (*P* = 0.001) and to Placebo (*P* = 0.041). The average score on the GAD-7 was 2.1 (1.2, 3.0) for Escitalopram, compared to 4.0 (3.0, 4.9) for Exercise and 3.1 (1.8, 4.4) for Placebo.

### 3.4. Depression

**Figure 2:**
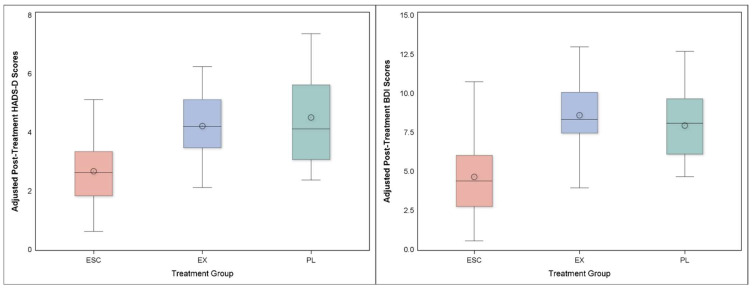
Comparison of scores on the HADS-D (left panel) and BDI-II (right panel) by treatment group at one year. Adjusted HADS-D and BDI-II Scores at 1-year follow-up revealed that participants in Escitalopram (ESC) had lower scores on the HADS-D (*P* = 0.004) and lower scores on the BDI-II (*P* = 0.004) compared to those participants randomized to Exercise (EX). It should be noted that depression scores were still lower for all groups compared to their pre-treatment levels for the BDI-II (baseline Exercise = 16.2; Escitalopram = 15.2; Placebo 17.7) and HAM-D (baseline Exercise = 6.4; Escitalopram 6.7; Placebo = 7.0). Multiple regression analyses were used with 1-year outcomes serving as the outcome variables for the HADS-D and BDI-II respectively, controlling for the pre-treatment level of the respective outcome, age, sex, race, baseline diagnosis of anxiety disorder, history of myocardial infarction, and treatment group as the predictor of interest.

For the HADS-D, we found no differences between the active treatment groups and Placebo (*P* = 0.172), while Escitalopram had lower scores compared to Exercise (*P* = 0.004) (Figure 2, left panel). Treatment group scores on the HADS-D were 2.8 (1.9, 3.7) for Escitalopram, 4.2 (3.2, 5.2) for Exercise, and 4.2 (2.8, 5.6) for Placebo (Figure 2; left panel). Participants in the Escitalopram group had lower HADS-D scores compared to Placebo (*P* = 0.021), while there was no difference between Exercise versus Placebo (*P* = 0.894). 

Similarly, we found no differences in BDI-II scores between active treatments and Placebo (*P* = 0.645), but Escitalopram had lower scores compared with Exercise (*P* = 0.004) (Figure 2, right panel). Treatment group scores were 8.2 (6.2, 10.2) for Exercise, 5.4 (3.6, 7.3) for Escitalopram, and 8.4 (5.6, 11.2) for Placebo.

### 3.5. Perceived Stress

No differences in PSS scores were observed for the active treatment groups versus placebo controls (*P* = 0.250), while those randomized to Escitalopram reported lower levels of stress compared to Exercise (*P* = 0.002) and Placebo (*P* = 0.026). Scores on the PSS scale were 16.2 (14.2, 19.0) for Escitalopram, 20.2 (17.8, 22.7) for Exercise, and 21.8 (18.2, 25.4) for Placebo. 

## 4. Discussion

One year after completion of the intervention, UNWIND trial participants who initially were randomized to Escitalopram reported less anxiety compared to those randomized to Exercise or to a Placebo. After 3 months of treatment, the average score on the HADS-A was 3.4 for Escitalopram, 5.2 for Exercise and 5.7 for placebo; 12 months following treatment, HADS-A scores for Escitalopram were 4.4 compared to 5.6 for Exercise and 5.2 for Placebo. These results confirm the efficacy of escitalopram for the treatment of anxiety in CHD patients and suggest that the benefits of escitalopram persist for a year.

Although there was reason to believe that exercise also may be beneficial in reducing anxiety in anxious individuals [25,26,27,28], results from previous trials have been inconsistent, and small samples and important methodological limitations raised concerns about the value of exercise for treating anxiety. Ramos-Sanchez and colleagues [28] identified 13 RCTs that met their eligibility criteria, comprising 731 adult participants. Exercise was considered to have ‘small/bordering medium’ anxiolytic effects in persons with anxiety and related disorders. However, of the 13 identified studies, only four studies (31%) reported a statistically significant difference between exercisers and controls. Additionally, Ramos-Sanchez advised caution in interpreting their results, identifying multiple serious methodological concerns across studies including randomization problems, selection of outcome measures, high degree of missing data, high attrition, and substantial risk of bias due to missing data in half of the studies. Further, small studies of patients with anxiety in other select populations including breast cancer [29], Parkinson’s Disease [30], and post-traumatic stress disorder [31], have produced null effects.

Although a number of studies have demonstrated the anti-depressant effects of exercise in patients with clinical depression [32] and in depressed patients with CHD [33], in the present study, exercise did not result in greater reductions in depression compared to placebo controls. The lack of difference between Exercise and Placebo is surprising, especially since the participants randomized to Exercise reported higher levels of physical activity at 1-year follow-up compared to those participants randomized to Escitalopram or Placebo. This apparent lack of greater benefit for exercise in reducing depression could be due to a floor effect (scores were lower relative to baseline but not lower compared to placebo), but also is consistent with findings that comorbid anxiety may attenuate the beneficial effects of exercise on depression [34].

The study has several limitations. First, the sample was relatively small and a third of the sample was lost to follow-up after a year. However, the pattern of results parallels the findings from immediate and 6-month post-treatment. Physical activity at follow-up was quantified by the Godin Leisure Time Physical Activity Questionnaire, an established and validated self-report instrument, but is subject to response bias and inaccurate self-report. Further, the longer-term benefits of escitalopram should be viewed with caution since 37% of patients randomized to Escitalopram reported taking psychotropic medications at 1-year follow-up compared to 17% in Exercise and 6% in Placebo. Although it is possible that the treatment group differences could be attributed to the greater ongoing treatment of anxiety among those patients randomized to escitalopram, treatment group differences were maintained when those patients reporting psychotropic medication use during the follow-up period were removed from the analyses. Thus, the results suggest that the benefits of escitalopram persist, even in those participants who discontinued their medication after the completion of the 12-week trial.

In summary, 1-year follow-up of the UNWIND trial revealed that treatment with escitalopram was associated with greater reductions in anxiety and depression compared to exercise and placebo controls. Escitalopram appears to be a safe and effective treatment of anxiety in anxious patients with stable CHD. Although exercise has many health benefits, it does not appear to be effective in treating anxiety, at least as measured by the questionnaires used in this study.

## Figures and Tables

**Table 1:** Demographic and clinical characteristics.

Treatment Group				
**Variable**	**Aerobic Exercise**	**Escitalopram**	**Placebo**	**Total Sample**
	**EX**	**ESC**	**PL**	
	**(*N* = 52)**	**(*N* = 53)**	**(*N* = 23)**	**(*N* = 128)**
***Demographic Characteristics***				
Age	65.2 (10.1)	63.9 (8.6)	65.2 (10.8)	64.6 (9.6)
Sex (Female)	17 (33%)	14 (26%)	6 (26%)	37 (29%)
Race				
Caucasian	39 (75%)	36 (68%)	18 (78%)	92 (72%)
African-American	8 (15%)	12 (23%)	5 (22%)	25 (20%)
Other	5 (10%)	5 (9%)	0 (%)	11 (8%)
Marital Status				
Married/ Co-Habiting	43 (83%)	41 (77%)	17 (74%)	98 (77%)
Single, Never Married	5 (10%)	3 (6%)	2 (9%)	10 (8%)
Divorced/Separated	4 (8%)	6 (11%)	2 (9%)	12 (9%)
Widowed	0 (0%)	3 (6%)	2 (9%)	5 (4%)
***Clinical Characteristics***				
Prior Myocardial Infarction	29 (56%)	31 (60%)	13 (57%)	73 (57%)
Prior Stent	37 (71%)	38 (73%)	16 (70%)	91 (71%)
Prior Cabg	12 (23%)	12 (23%)	6 (26%)	30 (23%)
Smoker, N (%)	2 (4%)	4 (8%)	4 (17%)	10 (8%)
Diabetic, N (%)	16 (31%)	19 (36%)	11 (48%)	46 (36%)
Clinic SBP, MM HG	127 (17)	126 (17)	128 (16)	127 (17)
Clinic DBP, MM HG	74 (9)	73 (9)	74 (11)	73 (9)
Total Cholesterol, MG/DL	157 (38)	152 (43)	150 (48)	154 (42)
Low Density (LDL), MG/DL	81 (33)	83 (37)	77 (39)	81 (36)
High Density (HDL), MG/DL	48 (14)	47 (12)	45 (14)	47 (13)
Very Low Density, MG/DL	27 (11)	23 (11)	28 (15)	25 (12)
Triglycerides	143 (74)	113 (56)	138 (74)	130 (68)
DSM 5 Diagnosis of Anxiety Disorder (Y/N), N (%)	37 (71)	37 (70)	19 (83)	93 (73)

## Data Availability

The data that support the findings of this study are available upon reasonable request.
